# Seasonal Mesophotic Coral Bleaching of *Stylophora pistillata* in the Northern Red Sea

**DOI:** 10.1371/journal.pone.0084968

**Published:** 2014-01-15

**Authors:** Orit Nir, David F. Gruber, Eli Shemesh, Eliezra Glasser, Dan Tchernov

**Affiliations:** 1 Marine Biology Department, The Leon H. Charney School of Marine Sciences, University of Haifa, Haifa, Israel; 2 Department of Natural Sciences, City University of New York, Baruch College, New York, New York, United States of America; 3 American Museum of Natural History, Sackler Institute of Comparative Genomics, New York, New York, United States of America; University of Sydney, Australia

## Abstract

Coral bleaching occurs when environmental stress induces breakdown of the coral-algae symbiosis and the host initiates algae expulsion. Two types of coral bleaching had been thoroughly discussed in the scientific literature; the first is primarily associated with mass coral bleaching events; the second is a seasonal loss of algae and/or pigments. Here, we describe a phenomenon that has been witnessed for repeated summers in the mesophotic zone (40–63 m) in the northern Red Sea: seasonal bleaching and recovery of several hermatypic coral species. In this study, we followed the recurring bleaching process of the common coral *Stylophora pistillata*. Bleaching occurred from April to September with a 66% decline in chlorophyll *a* concentration, while recovery began in October. Using aquarium and transplantation experiments, we explored environmental factors such as temperature, photon flux density and heterotrophic food availability. Our experiments and observations did not yield one single factor, alone, responsible for the seasonal bleaching. The dinoflagellate symbionts (of the genus *Symbiodinium*) in shallow (5 m) *Stylophora pistillata* were found to have a net photosynthetic rate of 56.98–92.19 µmol O_2_ cm^−2^ day^−1^. However, those from mesophotic depth (60 m) during months when they are not bleached are net consumers of oxygen having a net photosynthetic rate between −12.86 - (−10.24) µmol O_2_ cm^−2^ day^−1^. But during months when these mesophotic corals are partially-bleached, they yielded higher net production, between −2.83–0.76 µmol O_2_ cm^−2^ day^−1^. This study opens research questions as to why mesophotic zooxanthellae are more successfully meeting the corals metabolic requirements when Chl *a* concentration decreases by over 60% during summer and early fall.

## Introduction

Most scleractinian corals engage in an obligate mutualistic symbiosis with “zooxanthellae,” which are photosynthetic dinoflagellates, belonging predominantly to the genus *Symbiodinium* that reside in the animal's gastrodermal cells. The corals' success in shallow, tropical, oligotrophic marine environments is attributed to their association with these symbiotic dinoflagellates; evidence of this relationship exists for the past ∼251 million years [1]. In modern corals, the alga produces up to 95% of the corals' carbohydrates requirements [2] and algal photosynthesis has been reported to enhance calcification in the coral host [3] [4]. In deep dwelling corals, there is evidence that heterotrophic feeding supplies a large portion of the corals energetic demands [5].

Two types of coral bleaching have been thoroughly discussed in scientific literature. The most prominent type is associated with the exodus of the symbiotic algae from the host tissue via several triggering mechanisms [6]–[9]. This type of bleaching is commonly associated with global coral bleaching events that results in significant coral mortality [10], [11] and is typically correlated with increased water temperatures [12]. The second type of bleaching is a seasonal loss of algae and/or pigments- a dynamic bleaching that does not result in coral mortality and is mainly associated with light intensity shifts [13], [14]. It has been suggested that seasonal acclimation of coral may be caused by cyclical changes in the symbiotic algae community [15]–[18].

With the onset of technical diving in the Gulf of Eilat, northern Red Sea in 2004, there have been increased investigations into the Mesophotic Coral Ecosystem (MCE) areas that extends from 30 m to beyond 100 m and is characterized by the low availability of light for photosynthesis. In this MCE, we began observing a surprising phenomenon that occurs every summer: seasonal bleaching and recovery of several hermatypic coral species. The bleaching was most pronounced between 40 m and 63 m, the lower edge of the reef slope. Mesophotic coral bleaching has only been noted once before, in the Cayman Islands, down to a depth of 84 m [19] and was hypothesized to occur due to increased temperature and/or high Photon Flux Density (PFD).

This investigation is aimed at characterizing deep water bleaching of the key coral *Stylophora pistillata* as observed in the northern Red Sea, ranging from the shallow reef down to 63 m. We present a description of the dynamics of this phenomenon over the course of a year and examine the environmental factors surrounding the mesophotic bleaching. We measured main physiological parameters, describing photosynthesis and respiration rates seasonally. This is a first attempt to separate out the primary factors that possibly lead to seasonal “bleaching” in a deep coral ecosystem, using both tank and transplantation studies in conjunction with long-term environmental monitoring data.

## Materials and Methods

### 1. Study site and sample collection and handling

All research described here was conducted at the Gulf of Eilat, Israel (29°30′06.8″ N 34°55′02.8″ E). Survey and replicate collection was carried out along the continuous slope (0–63 m) in front of the Inter-University Marine Institute (IUI) using open water SCUBA (TRIMIX protocol). In October 2010, we marked 10 *Stylophora pistillata* colonies at depth 55–60 m. These were revisited every month for the following year, and the percent of colony surface area that was bleached was measured by placing a 50×50 cm^2^ frame, subdivided by nylon string to 5×5 cm^2^ squares and counting bleached/unbleached squares according to visual estimation.

*S. pistillata* fragments of ∼5 cm length were collected on five different occasions from the deep reef at 55–60 m (September and October 2010, March, May, and September 2011) and twice from shallow reef at 5 m (October 2010 and May 2011) for several experiments, as detailed below. The collection was conducted under permit number 31944 from the Israel Nature and Parks Authority. Each month, at each depth, fragments were collected from five different colonies in a black collection basket that prevents exposure to sunlight. Fragments were then transferred to outdoor water tables (no artificial light) with PFD sets that corresponded to those found at 60 m and 5 m at the month of collection. Ambient PFD (September: ∼40 µmol quanta cm^−2^ s^−1^, October:∼15 and 1000 µmol quanta cm^−2^ s^−1^, March:∼60 µmol quanta cm^−2^ s^−1^, and May: 20 and 1170 µmol quanta cm^−2^ s^−1^) was calibrated from data from the International Monitoring Program in the Gulf of Eilat (http://www.iui-eilat.ac.il/NMP/Default.aspx) as well as other local measurements [20]. These PFDs were obtained by applying filters (Lee Filters; # 172: Lagoon Blue) above and around the tanks, also blocking UV radiation. The water tables received continuous water flow from seawater pumped from 30 m that varied ±0.5°C daily.

### 2. Experiments

#### Aquarium experiments

**Figure 1:**
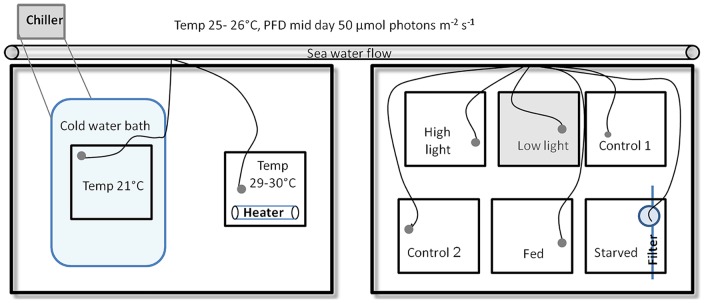
Experiment setup. Black squares represent the two water tables, holding the eight experiment tanks and experiment systems.

Aquarium experiments were performed in 10 liter tanks on fragments of partially bleached *S. pistillata* from 60 m and were conducted between September-October, 2011(Fig. 1). Five colonies were taken out of the mesophotic reef and, on the same day, fragmented and distributed evenly in eight experimental tanks submerged in water tables, one fragment from each colony in each treatment (5×8 = 40 fragments), The experiment began on the following day with a 10-day acclimation period, conditions in the tank were as described above. On the 11^th^ day, each tank was set to one of six experimental conditions These included: 1) High temperature (29–30°C); 2) Low temperature (21°C); 3) High PFD (∼135 µmol quanta cm^−2^ s^−1^); 4) Low PFD (∼20 µmol quanta cm^−2^ s^−1^); both measured with Li-Cor (LI-190) quantum sensor at surface; 5) starvation (0.2 µm filtered seawater); and 6) heterotrophically-fed (feeding once/day with the brine shrimp, *Artemia nauplius* (50 ml of 75 *Artemia*/ml^−1^). Over the next 12 days, fragments were assessed every three days for photosynthetic and respiration rates in a metabolic chamber as described below. On day 23, tissue and algae of the coral fragments were removed as described below.

#### Transplantation experiments

Large *S. pistillata* fragments (15 cm length) taken from the same colonies used in the tank experiments (two fragments from each colony, total of 10 fragments), were transplanted the following day after being taken out of the reef (September 2011) to 30 m on an underwater table positioned on the reef slope. Transplants were visited 10 days following transplantation and appeared completely bleached, though still living. On the 23^rd^ day, coral fragments were collected for algae and chlorophyll analysis as described below, one fragment from each original colony (five fragments).

### 3. Laboratory analysis

In September and October 2010 and in March and May 2011, fragments were taken for a simultaneous continuous measurement of chlorophyll fluorescence and oxygen production and consumption (60 m and 5 m fragments, as described above). Measurements were taken within 24 hours of collection. Fragments were measured in a metabolic chamber, submerged in 0.2 µm filtered seawater and stirred continuously. Temperature was 22–26°C, similar to the reef at the time and depth of collection; it was documented by a thermometer mounted on the oxygen optrode (FDO 925, WTW). PFD was measured with a spherical micro quantum sensor (US-SQS, WALZ) connected to the DUAL-PAM/F analyzer and software (WALZ) along with an optical fiber supplying measuring light and saturating pulses, and documenting the fluorescence signal at 685 nm. Actinic light was provided by a double fiber light source (CL 1500 ECO, SteREO, ZEISS).

Each fragment was subjected to one measurement conducted as follows: 10 min dark acclimation+measurement of oxygen followed by continuously increasing actinic PFD (10 min for each PFD, 10–11 levels of PFD from 2 µmol quanta cm^−2^ s^−1^ to 1800 µmol quanta cm^−2^ s^−1^). Continuously increasing the actinic light over 2 hours aimed to imitate the conditions on the reef. After the experiment, each fragment was placed in liquid nitrogen and placed in −80°C until further analysis: Coral tissue was removed with an air brush and tissue was analyzed for algae concentration via direct counts under a light microscope using a C-ChipTM hemocytometer; chlorophyll *a* concentration and fragment surface area were recorded as described by [21], [22]. A small portion from each sample was taken for phylogenetic identification of the symbiotic algae.

### 4. DNA amplification and taxonomic identification

Zooxanthellae DNA was extracted using a protocol described by Nir [23], followed by PCR amplification of the ITS-2 region with the primers “ITSintfor2” (5′GAATTGCAGA ACTCCGTG-3′) and “ITS2CLAMP” (5′ GGGATCCATATGCTTAAGT TCAGCGGGT-3′) [24]. PCR products were cleaned (Wizard SV Gel and PCR Clean-Up System, Promega), and ligated into the pGEM vector (Promega, Madison, WI). The plasmids were inserted into competent *Escherichia coli* cells (strain DH5) and cultured on Fast Media Lab Agar IPTG/X Gel plates (Fermentas, Vilnius, Lithuania). We sequenced the ITS2 rRNA gene (337 bp) from 23 deep colonies (6–10 clones/colony, totaling 172 sequences) and 5 shallow colonies (9–10 clones/colony, totaling 49 sequences) (HyLabs, Israel). Sequences were viewed and analyzed with BioEdit software.

### 5. Data analysis

The following photosynthetic parameters were calculated from oxygen measurements, normalized both to chlorophyll *a* concentration and coral surface area, by using the hyperbolic tangent function in Excel: respiration (R), maximum rate of photosynthesis (Pmax), maximum rate of photosynthesis under limited light conditions (α), and PFD at photosynthetic- respiration compensation point (Ek). The parameter Effective PSII quantum yield (YII) was calculated from chlorophyll fluorescence data according to Genty et al. [25], who refer to it as ΔF/Fm′. One and two way ANOVA statistical analysis was calculated with SigmaPlot software, after performing normality and equal variance tests. Pairwise multiple comparison procedures were calculated with Holm-Sidak method to an overall significance level of p≤0.05. Data from the International Monitoring Program in the Gulf of Eilat was downloaded from their database at http://www.iui-eilat.ac.il/NMP/database/database.aspx and analyzed with SigmaPlot software.

## Results

**Figure 2:**
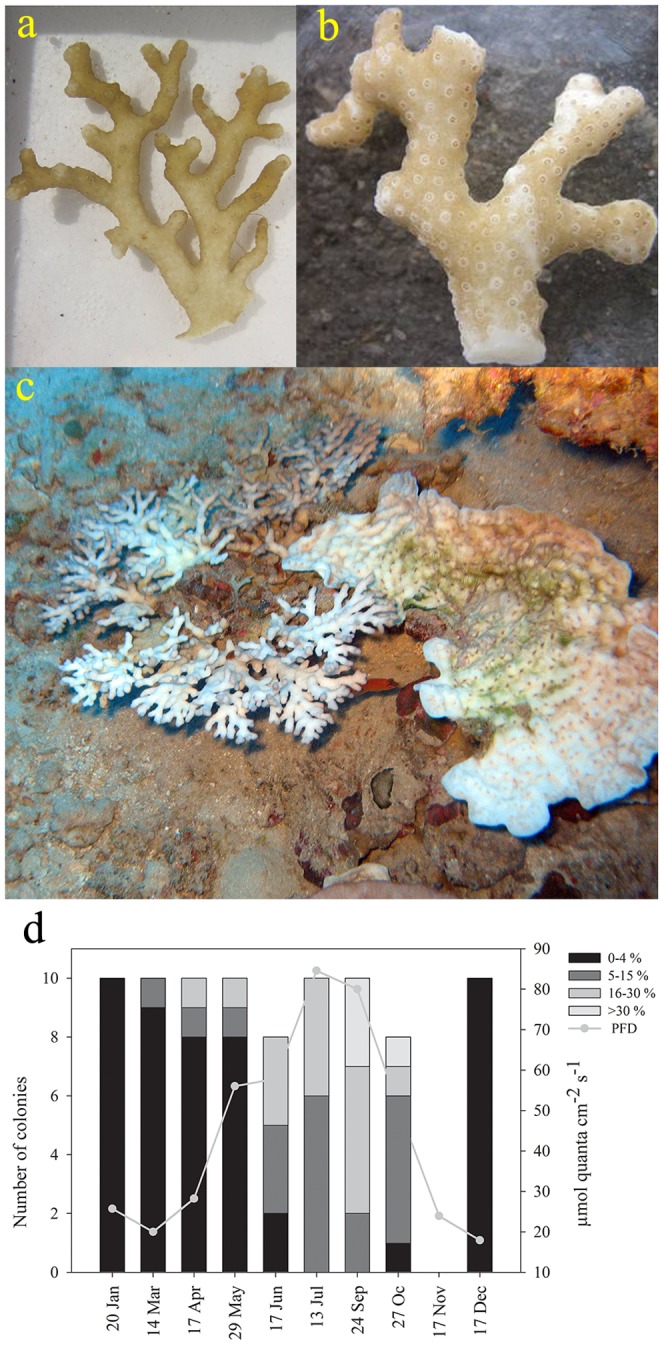
*S. pistillata* bleaching at 60 m depth. (a) Unbleached fragment during March 2010. (b) “Chicken pox” bleaching pattern during October 2010. (c) On the left, *S. pistillata*, on the right *Mycedium sp.* both bleached and alive, during September 2011. (d) Percentage of bleaching measured for 10 colonies in 60 m, surveyed along 2010. Colors represent percentage of bleaching; lighter colors represent higher parentage of bleaching. Gray line indicates average monthly PFD at midday.

We followed the recurring bleaching and recovery process of the coral *Stylophora pistillata* in the mesophotic reef for one year (September 2010–September 2011). *S. pistillata* colonies present a range of pigment shades during the months of June to November, consisting of unbleached (Fig. 2a), moderately and unevenly bleached (Fig. 2b), and in a few cases, entirely bleached (Fig. 2c). The anatomical distribution of algae pigmentation in *S. pistillata* is uneven, with the area around the polyp (around the septa) losing almost all its color, and the polyps' tentacles and inter-polyp areas (coenosarcs) showing little to no loss of color (Fig. 2b), creating a spotted pattern.

**Figure 3:**
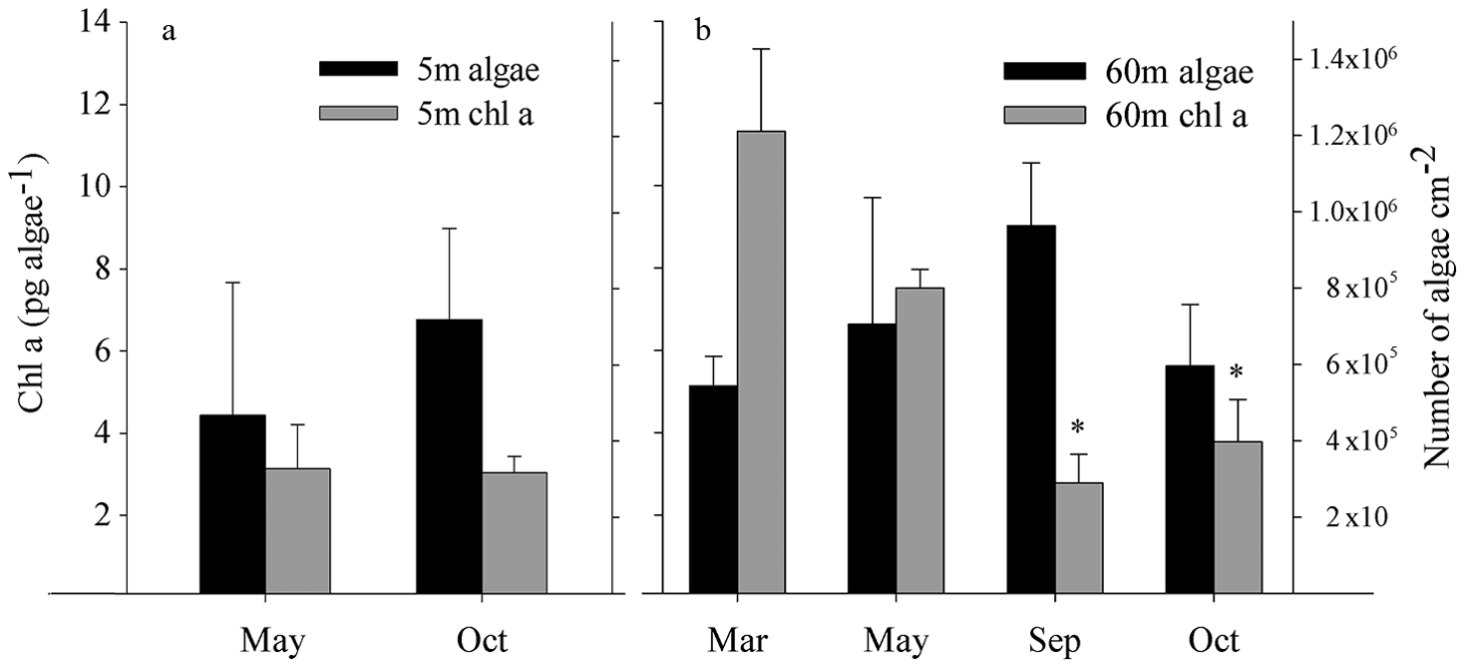
Algae numbers and chlorophyll concentration. Algae numbers (black) and Chl *a* concentration per algae (gray) for *Symbiodinium sp.* within *S. pistillata* (a) Collected at 5 m depth during May and October (b) collected at 60 m, September and October represent bleached and partially bleached fragments; March and May are not bleached. Scales of both y-axes in figures (a) and (b) are similar for ease of comparison. n = 5 per each month in each depth, bars represent standard deviation.

**Figure 4:**
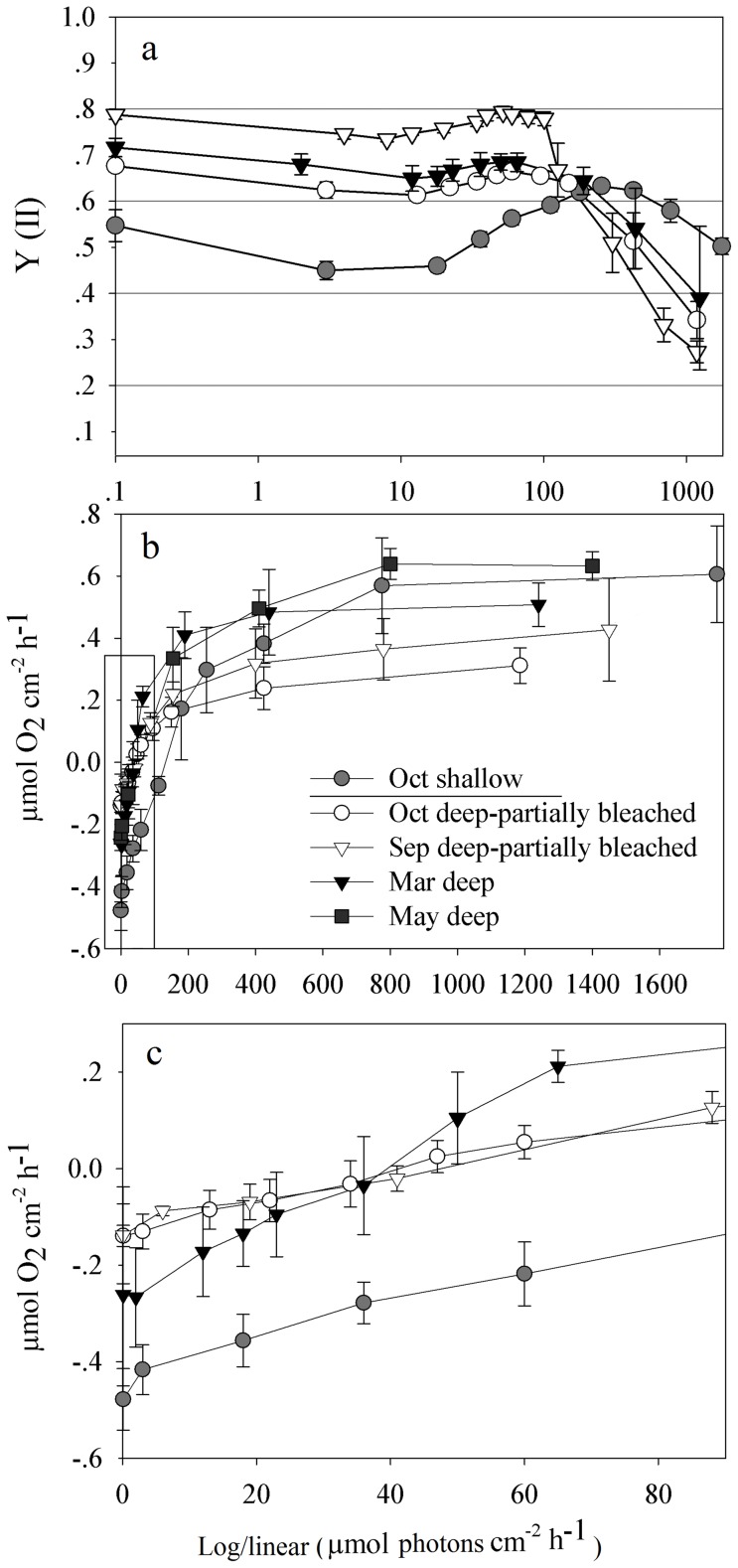
Photosynthesis and respiration rates for *S. pistillata* from 60 m. Chl *a* fluorescence yield (a) and O_2_ production/consumption (b, c), plotted to light intensity (FI curve and PI curve, respectfully) for *S. pistillata* from 60 m. October and September represent bleached and partially bleached colonies, March and May are not bleached. A shallow corals plot is added for comparison. n = 5 fragments from different colonies per each line. Legend for the three plots is in Figure 4(b). Bars represent standard deviation. (b) O_2_ production/consumption plotted to light intensity (PI curve). Red rectangle includes PFD that is ecologically relevant for 60 m colonies and is enlarged in Fig. 4(c). (C) An enlargement of the low PFD area in the PI curve, relevant for 60 m. For better appreciation of the results, only selected fragments are presented.

Recovery begins in October–November and full recovery is obtained by December (Fig. 2d). The cause for the apparent color change of the colonies is a yearly change of 66% in chlorophyll *a* concentration normalized by algal cell (Fig. 3, ANOVA's p<0.001, Holm-Sidak method distinguishes October and September from March and May, p = 0.05), similar results were obtain when normalizing chlorophyll *a* to coral surface area (October- 2.2±0.6 and March- 6.05±0.5 pg Chl *a* cm^−2^, data not shown). Algae numbers did not change significantly between the seasons (Fig. 3, one way ANOVA, p = 0.13) even though the mean algae density increased over this period, the differences were not significant. Fluorescence Irradiance (FI) measurements of photosynthetic yield, from 60 m showed no significant difference between unbleached and pigment bleached fragments (Fig. 4a and Table 1). Photosynthetic Irradiance (PI) curves (Fig. 4b, 4c) yield the following parameters: rate of photosynthesis under limited light conditions (α), maximum photosynthetic rate (Pmax), compensation PFD (Ek) and dark respiration (R) all being higher in unbleached corals (Table 1). Integration of net daily O_2_ production/consumption (µmol O_2_ cm-^2^ Day-^1^) yields negative results during March and May and positive results in September and October (Table 2).

**Table 1:** Common photosynthetic physiological parameters, presented for deep (60 m) bleached and unbleached and shallow (5 m) *S. pistillata* corals.

Parameter/Coral type	March 60 m	May 60 m	May 5 m	September 60 m	October 60 m	October 5 m	ANOVA (for deep fragments)
Photosynthetic yield during the dark (YII)	0.71±0.02	0.87±0.047	0.60±0.19	0.78±0.009	0.67±0.011	0.54±0.034	tow way ANOVA for between month p = 0.006, between llight intensity p<0.001.
Rate of photosynthesis under limiting light conditions (α)	0.0054±0.0007	0.0036±0.001	0.0026±0.0003	0.0028±001	0.0033±0.00005	0.034±0.009	ANOVA p<0.001, Holm-Sidak method significantly distinguishes Mar from Oct, Sep and May (p<0.01)
Maximum photosynthetic rate (Pmax), µmol O_2_ cm^−2^ h^−1^	0.51±0.07	0.63±0.05	0.79±0.09	0.43±0.17	0.31±0.05	0.61±0.16	ANOVA p<0.001, Holm-Sidak method significantly distinguishes Oct from Mar and May and Sep from May (p<0.01)
Compensation PFD (Ek), µmol quanta cm^−2^ s^−1^	53±13	65±6	265±45	44±3	39±7	140±12	ANOVA p<0.001, Holm-Sidak method significantly distinguishes Oct from Mar and May and Sep from May (p<0.01)
Dark respiration (R), µmol O_2_ cm^−2^ h^−1^	−0.26±0.19	−0.24±0.04	−0.77±0.08	−0.14±0.1	−0.14±0.02	−0.48±0.06	ANOVA p = 0.018, Holm-Sidak method significantly distinguishes Oct and Sep from Mar and May (p≤0.05)
Chl *a* concentration, pg/algae	10.13±2.00	7.51±0.46	3.13±1.08	2.78±0.78	3.78±1.03	3.04±0.40	ANOVA's p<0.001, Holm-Sidak method distinguishes Oct and Sep from Mar and May, p = 0.05

**Table 2:** Net daily O_2_ production/consumption (µmol O_2_ cm^−2^ Day^−1^) for shallow and mesophotic *S. pistillata* corals.

	March	May	September	October
5 m	No data	92.19	No data	56.98
60 m	−12.86	−10.24	0.76	−2.83

**Figure 5:**
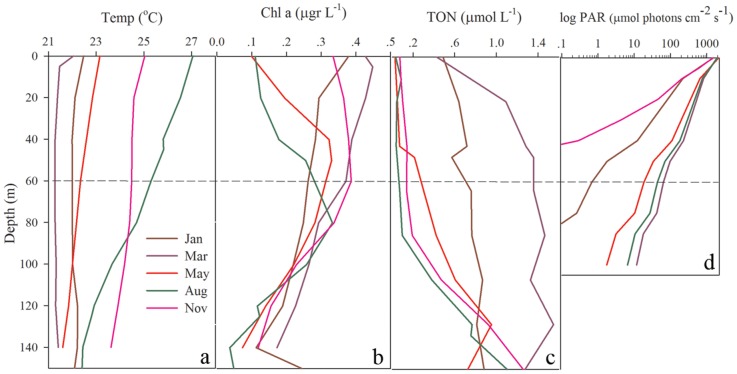
Environmental gradients in the north Red Sea water column. The environmental parameters (a) temperature, (b) Chl *a*, (c) total organic nitrogen and (d) logarithm of PAR in the Gulf of Eilat. Parameters are plotted along a 140 m depth gradient, during March (red), May (green), August (blue) and November (brown). Dotted line highlights 60 m depth.

**Figure 6:**
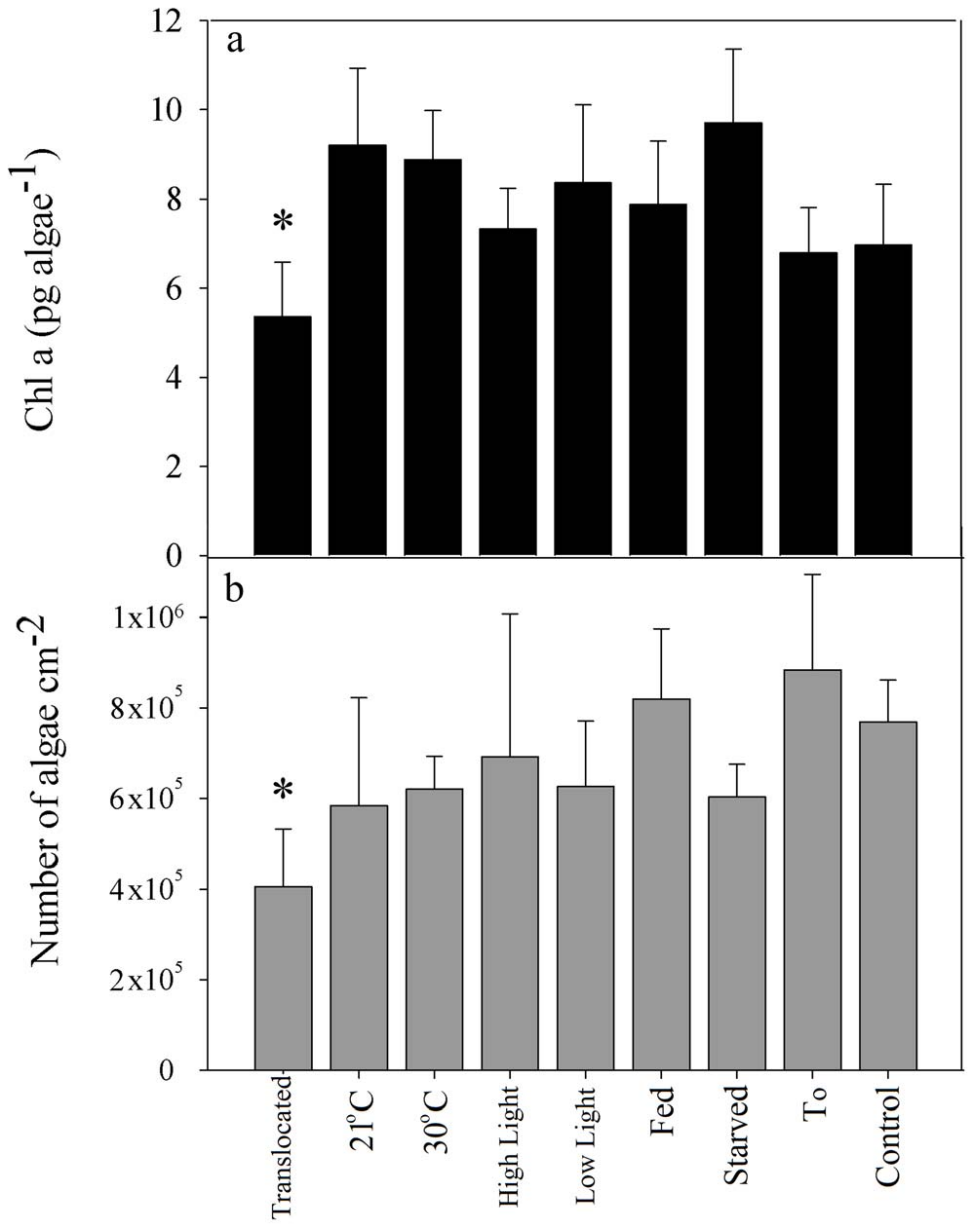
Results of tank experiment. (a) Chl *a* concentration per algae (in black) and (b) algae numbers (in gray), of *S. pistillata* subjected to six different treatments (chilling the tank water to 21°C, hitting the tank water to 29–30°C, feeding once a day with *Artemia nauplius*, filtration of sea water via 0.2 µm mash creating conditions of starvation, High PFD of ∼135 µmol quanta cm^−2^ s^−1^ and Low PFD of ∼20 µmol quanta cm^−2^ s^−1^), T0 represent a measurement taken on the day the fragments were plucked, “Ctrl” is the control and “Trans” are the fraq1agments from transplantation experiment. n = 5 fragments per each treatment, control treatment includes 10 fragments. Bars represent standard deviation.

The International Monitoring Program in the Gulf of Eilat has been recording environmental data (such as temperature, light intensity, nitrate NO_3_^−^, nitrite NO_2_^−^ and chlorophyll *a*) since 2004 (http://www.iui-eilat.ac.il/NMP/Default.aspx). In the winter months (December-March), the water column cools to 21–22°C, leading to deep 300–700 m mixing and upwelling of nutrients. Summers are warm (∼27°C at surface ∼26°C at 60 m and ∼24°C at 140 m) with strong thermal stratification of the water column and exhibit a depletion of bionutrients in the photic zone (Fig. 5). The seasonal temperature variation induces many environmental changes that correlate with the yearly dynamic of zooxanthellae pigment bleaching. Using tank experiments, we explored the following environmental factors: temperature, photon flux density and heterotrophic food availability. However, none of these factors singlehandedly resulted in significant pigment loss (Fig. 6) even when considering colony genotype as a factor in ANOVA, the difference between treatments was still not evident (treatment: F = 2.261, p = 0.053, genotype: F = 0.966, p = 0.441). The transplanted fragments did undergo a significant decrease in chlorphyll *a* (−2.3±1.1 pg alga-1, p<0.001) and algae concentration (−4.1*10^5^ algae cm^−2^, p<0.001) after 23 days (Fig. 6).

Based on sequence data, algal communities from 60 m can be grouped into 12 distinct groups, each containing identical sequences and differing from each other by 1–10 bp. All sequences belong to clade C1 with the most similar GenBank sequence (NCBI BLAST, max indent of 97%, E value of 2e-155) being a deep dwelling coral (EU703806.1). The shallow algae community of *S. pistillata* contains only one type of clade A4. The deep algae community remains stable along the year and we found no correlation between the algae community within a coral and the month of sampling, or the bleaching status of the host. GenBank accession numbers of the 12 sequences: KC146408- KC146410 and KF111701- KF111709.

## Discussion

### 1. Mesophotic Bleaching

Here, we report for the first time a phenomenon that occurs every summer in the mesophotic zone (40–63 m) in the northern Red Sea: seasonal chlorophyll *a* bleaching and recovery of the hermatypic coral *Stylophora pistillata*. We describe its dynamics over a course of a year, revealing pigment bleaching that occurs along the spring and summer (April–September) and begins to recover during October (Fig. 2).

We explored what environmental factors may cause the bleaching. The monthly Monitoring Program in Gulf of Eilat allows us to rebuff possible bleaching causes, such as pollution (both organic and inorganic), extreme temperature, and fresh water lances, as well as to investigate yearly environmental changes that may induce bleaching. The temperature at 60 m depth in the Gulf of Eilat ranges during the year between 21°C–26°C (Fig. 5). These temperatures have not been reported to cause severe pigment bleaching responses [13], [14], [26], [27]. Further, in the aquarium experiments, fragments placed in 29–30°C did not experience pigment loss, suggesting that temperature alone is not the primary cause of the mesophotic pigment bleaching. Host starvation has been reported to result in lower zooxanthellae chlorophyll concentrations. When starved *S. pistillata* corals were compared to fed corals in the same light regime, chlorophyll *a* declined after 10 days in low PAR intensity [28], although Borell and Bischof [29] reported a change in chlorophyll *a* in S. *pistillata* starved and exposed to elevated temperature for 15 days. Starvation as a cause for mesophotic bleaching was tested in our aquarium experiment, but yielded no significant results (Fig. 6). The mesophotic zooxanthellae genotyping indicate no correlation between the algae community within a coral and the month of sampling or the status of pigment bleaching. Hence, phylogenetic data did not support the premise that pigment bleaching is related to symbiotic algae community change.

In the 60 m to 30 m transplantation experiment, a change of temperature (1°C increase from 25.8°C to 26.8°C), light intensity (from 14 µmol quanta cm^−2^ s^−1^ at 60 m to 162 µmol quanta cm^−2^ s^−1^ at 30 m) and spectrum (from 395–520 nm at 60 m to 340–589 nm at 30 m) (data not shown) caused a significant loss of pigments and algae. As the change in temperature is only 1°C, the dramatic increase in PFD (Fig. 5) likely played a role in bleaching the transplanted corals. Although in the tank experiment, varying light intensity alone (135 *vs.* 20 µmol quanta cm^−2^ s^−1^) did not cause pigment bleaching or bleaching associated with algae loss. Also, the UVA and UVB intensity in 30 m and 60 m is small (UVA 7 and 0.14 µmol quanta m^−2^ s^−1^ and UVB 0.6 and 0.03 µmol quanta m^−2^ s^−1^ of surface irradiance, respectively). The intensity described in the literature does not test affects of such low intensities. However, this bleaching behavior was composed of losing both chlorophyll and algae, unlike the mechanism of mesophotic bleaching.

### 2. Coral- Algal Symbiosis

Characterization of some photosynthetic parameters of the zooxanthellae reveals that the pigment bleaching decreases their photosynthetic rates (Table 1). With pigment bleaching occurring throughout a portion of the year (1–6 months) and with a corresponding reduction in photosynthetic rates, the energetic budget of the holobiont is likely affected. We integrated the photosynthetic-PFD production curve (Fig. 4b) with monthly PFD data at 60 m and 5 m (Monitoring Program in the Gulf of Eilat, http://www.iui-eilat.ac.il/NMP/Default. aspx) to produce an assessment of daily O_2_ production/consumption. Nighttime respiration was not included since it is an order of magnitude lower than daytime respiration. For example, in October, for 60 m colonies, we found average consumption of −0.0036±0.0011 µmol O_2_h^−1^chl *a* ^−1^ at night vs. −0.066±0.048 µmol O_2_h^−1^chl *a* ^−1^ of day respiration. This calculation has its limitations, mainly due to the approximated conditions of the metabolic chamber experiment, resembling, but not identical to the conditions in the reef. Still, we believe the trends presented in Table 2 are correct; during most of the year, the deep zooxanthellae do not perform photosynthesis at a rate necessary to fulfill even the respiratory demands of the coral holobiont. The comparison of the 60 m corals to 5 m corals is striking. The shallow algae are able to supply the host its carbon demands for respiration and growth, as shown before [30], yet deep corals need to rely on other carbon sources to satisfy their year-round respiratory and growth demands [5], [31].

Although the photosynthetic efficiency (α) is greater in March (0.0054±0.0007) than in September and October (0.0028±0.001, 0.0033±0.0005 respectively), the light intensity at that depth never exceeds the compensation point during March. The same applies to May. In September and October the compensation point is lower than in May and March due to reduced respiration rate. Combined with higher maximum PFD during these months, the zooxanthellae satisfy or are close to satisfying the basic metabolic demands of the coral, even thou Chl *a* concentration is low (2.78±0.69 and 3.78±1.03 pg/algae for September and October respectively).

Our results show that mesophotic zooxanthellae are not meeting the corals metabolic demands when Chl *a* concentration is high (7.51–10.13 pg/algae), but succeed in meeting its metabolic requirements when Chl *a* concentration is 60% lower in the summer and early fall. This indicates that seasonal bleaching of these mesophotic corals can be energetically beneficial for the coral holobiont and perhaps may be a part of a mesophotic coral's acclimation response to a seasonally changing environment [13], [14].

The coral-algal relationship is considered mutualistic relationship, however, it has been suggested that it can exist in a form where the coral is parasitic [32], [33] or vice versa, with the algae being parasitic [31], [34]. Previous work has even suggested that in mesophotic corals, carbohydrates are translocated from host to algae [5], [31]. Black antipatharian corals collected in extremely low light environments (as deep as 396 m) have also been shown to contain endosymbiotic *Symbiodinium* [35]. This study presents the possibility that there may be an oscillation in the mesophotic coral-algae relationship from mutualistic relationship in the summer to parasitic in the winter.
